# Effect of Bio-Cementation Level and Rainfall Intensity on Surface Erosion Resistance of Biotreated Slope Using PEICP Method

**DOI:** 10.3390/ma18071662

**Published:** 2025-04-04

**Authors:** Yuyuan Chen, Hemanta Hazarika, Nadella Marchelina

**Affiliations:** 1 Department of Civil Engineering, Kyushu University, Fukuoka 819-0395, Japan; chen.yuyuan.137@s.kyushu-u.ac.jp; 2 Department of Building Materials Engineering and Geoengineering, Lublin University of Technology, 20-618 Lublin, Poland; nadellamarchelina@gmail.com

**Keywords:** biomineralization, rainfall erosion, surface strength, soil loss

## Abstract

Biomineralization technology is a promising method for soil cementation, enhancing its mechanical properties. However, its application in mitigating slope surface erosion caused by rainfall has not been fully explored. This study experimentally examined the feasibility of using plant-based enzyme-induced carbonate precipitation (PEICP) to reduce slope surface rainfall erosion through simulated rainfall tests. The effects of biotreatment cycles (*N*) and rainfall intensity (*R*_i_) on erosion resistance were evaluated. The results demonstrated that increasing the biotreatment cycles improved the bio-cementation level, as evidenced by enhanced surface strength, increased calcium carbonate content (*CCC*) and thicker crust layers. Specifically, as the biotreatment cycles (*N*) increased from 2 to 6, the crust layer thickness expanded from 5.2 mm to 15.7 mm, with surface strength rising from 38.3 kPa to 244.3 kPa. Likewise, the *CCC* increased significantly from 1.09% to 5.32%, further reinforcing the soil structure and enhancing erosion resistance. Slopes treated with six biotreatment cycles exhibited optimal erosion resistance across rainfall intensities ranging from 45 to 100 mm/h. Compared to untreated slopes, biotreated slopes showed significant reductions in soil loss, with a decrease to below 10% at *N* = 4 and near-complete erosion resistance at *N* = 6. These findings highlight the potential of PEICP technology for improving slope stability under rainfall conditions.

## 1. Introduction

Due to the disturbance and altering in soil structure caused by human activities, natural and artificial slopes are more fragile in response to extreme weather [1,2]. Among the numerous existing slope degeneration problems, surface soil erosion has been a main concern in the domain of geotechnical engineering [3,4]. As one of the frequent types of soil degradations, rainfall-induced erosion should be taken seriously, thereby reducing the occurrence of artificial slope collapse and landslides [4,5,6,7,8]. To strengthen the slope stability and mitigate the soil erosion issue, multiple countermeasures including engineering protection with concrete and stone [9], geotextiles [10], geocell [11], plant protection [12,13,14] and chemical method [15] have been adopted far and wide. However, it should be noted that the above-mentioned methods have the shortcoming of ecosystem destruction, prohibitive cost, elaborate technology and environment restriction. Consequently, it is essential to develop an exploitable approach to tackle the slope surface erosion conundrum.

Currently, biomineralization technology is proposed as a new developing and eco-friendly approach which has enormous utilization potentiality in the domain of geotechnical engineering [16,17,18,19]. To realize the biomineralization process, ureolytic bacteria or urease participate in the hydrolyzation of urea, thereby producing carbonate ions. When a calcium ion is introduced by adding calcium salt, the carbonate ions bind with calcium ion and bring out the production of calcium carbonate precipitation, which plays the role of pore filling, particle roughening and interparticle binding in the soil matrix [20,21]. In accordance with the type of biocatalyst, biomineralization technology chiefly involved in the processes of microbially induced carbonate precipitation (MICP) and enzyme-induced carbonate precipitation (EICP) [22,23,24,25,26,27,28].

So far, substantial studies related to alleviating the erosion induced by the water from natural environment have been carried out. Via the approach of MICP or EICP, scholars performed a series of indoor experiments and large-scale field experiments to investigate the realistic possibility of enhancing the erosion resistance to sea wave [29,30,31,32,33,34] and internal seepage [35,36,37,38]. With the aid of simulated rainfall tests, the rainfall erosion resistance of biotreated slope model by means of MICP method [4,14,39,40,41,42,43,44] or EICP method [9,45,46] was inspected. To be specific, by conducting simulated rainfall erosion tests, microbial calcification has been shown to effectively rainfall erosion control on sandy slope [4], loess slope [14,41], silt slope [39], slope with various angles and relative density [40], sandy slope with different gradations [42], field scale slope [43] and granite residual soil slope [44]. According to this, the application of EICP method in slope surface erosion resistance to rainfall is relatively limited. He et al. [9] examined the use of EICP combined with fibers to improve clay soil slopes, focusing on erosion caused by rainfall and surface runoff. Sun et al. [45] applied enzymatic calcification to enhance dust control and rainfall erosion resistance, showcasing its effectiveness in reducing erosion. Sun et al. [46] further explored the durability of enzymatically induced carbonate precipitation, highlighting its enhanced resistance to rainfall erosion.

It is worth mentioning that the biosafety issue has been a major concern when popularizing MICP technology from the laboratory to practical engineering [47,48,49,50,51]. Moreover, it is reported by Meng et al. [52] and Wang et al. [21], that, compared with the MICP method, the EICP method have superiority of low technical threshold and greater application sphere. However, commercial pure urease was commonly utilized in previous studies, which limits the large-scale promotion of EICP technology [53,54,55,56]. When utilizing the urease extracted from soybean as biocatalyst in the biomineralization process, the above-mentioned issue can be solved on target [21,57,58,59,60,61,62,63]. In nature, soybean urease enzyme-induced calcium carbonate precipitation method can be classified as the category of plant-based enzyme-induced calcium carbonate precipitation (PEICP), which embodies better advantage of low cost and high popularization prospect.

The primary purpose of current study is to experimentally examine the feasibility of utilizing the PEICP method as a slope surface rainfall erosion countermeasure. Small-scale slope models were biotreated via soybean urease enzyme-induced calcium carbonate precipitation process and subjected to simulated rainfall tests. Different from previous studies only considering single rainfall intensity [4,9,14,39,41,42,43,44,45,46], this study further comprehensively takes into account the influence of different rainfall intensities *R*_i_ (45, 70, 100 mm/h) and different bio-cementation levels (biotreatment cycles *N* of 2, 4, 6) on the rainfall erosion resistance of biotreated slope models. Meanwhile, comparison was made in terms of the surface strength, thickness of crust layer, calcium carbonate content (*CCC*), visual erosion observation, percentage of accumulative soil loss weight and erosion rate. Furthermore, microscope analysis encompassing scanning electron microscopy (SEM) tests and X-ray diffraction (XRD) analysis were conducted to disclose the essential mechanism of surface erosion control via PEICP approach.

## 2. Materials and Methods

### 2.1. Materials

**Figure 1:**
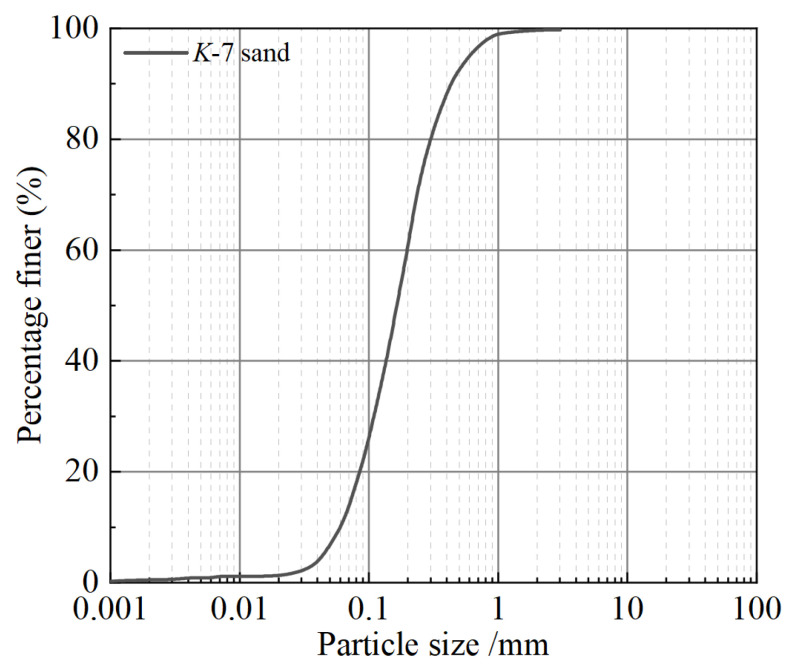
Grain-size distribution curve for the silica sands *K-7*.

To induce the biomineralization process, biotreatment solutions, including soybean crude urease solution (SCUS) and cementation solution (CS), were prepared. The soybean crude urease, acting as the biocatalyst in the process of urea hydrolysis, was derived by referring to the extraction procedure from prior studies [21,52,63,64,65]. In this study, medium-grain soybeans (food-grade, non-GMO), produced in Hokkaido, Japan, were purchased from the Asahishokuhinkogyo Co., Ltd. (Nankoku, Japan), and used as raw material. By using grinder, the dry soybean bought from market was smashed into powder. Then, the soybean powder was sieved through a screen with a pore diameter of 0.25 mm. A certain mass of sieved soybean powder was mixed uniformly with distilled water using magnetic stirrer for 15 min, during which the soybean crude urease was extracted from soybean powder to turbid liquid. Subsequently, the turbid liquid was further centrifuged I confirmand filtered to obtain the clear liquid, which contained soybean crude urease. By accomplishing the above-mentioned procedures, the stand-by soybean crude urease solution (SCUS) was prepared. Note that, in the current study, 40 g sieved soybean powder was added into 1 L distilled water to obtain the SCUS with the concentration of 40 g/L. In reference to Cui et al. [48] and He et al. [9], a cementation solution (CS) encompassing equal mole concentration (0.5 mol/L) of urea and CaCl_2_ was applied in this study to assist the process of biomineralization. The test soil used to prepare the slope model was *K*-7 standard testing silica sand, which is from Kumamoto, Japan. The basic properties of *K*-7 sand are shown in Table 1. The grain-size distribution curve for the silica sands *K*-7 is shown in Figure 1.

### 2.2. Test Arrangement

**Figure 2:**
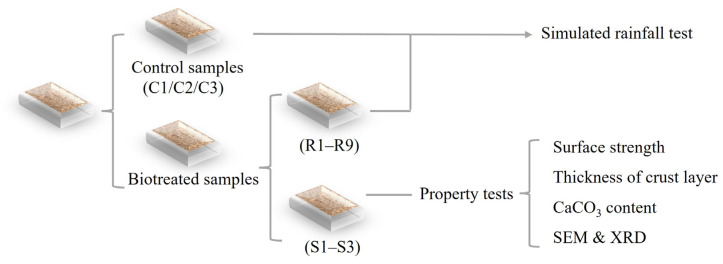
Schematic diagram of experimental plan for slope model.

The slope model arrangements are summarized in Table 2. A total of 15 models, subjected to different biotreatment cycles and rainfall intensities, were prepared for this study. The different bio-cementation levels were achieved by applying varying biotreatment cycles (2, 4, and 6) to the slope models. Additionally, for each biotreatment cycle, the rainfall erosion resistance of the models was tested under different rainfall intensities, as shown in Table 2. The control group consisted of slope models (C1, C2, and C3), which were subjected to simulated rainfall tests at different rainfall intensities to observe the erosion conditions of untreated slope models. These models were not subjected to biotreatment. Models (S1, S2, and S3) were used solely for property tests, including the measurement of surface strength, crust layer thickness, and calcium carbonate content (*CCC*), as well as conducting scanning electron microscopy (SEM) and X-ray diffraction (XRD) analyses. A schematic diagram of the experimental plan for the slope models is shown in Figure 2.

### 2.3. PEICP Biotreatment for the Small-Scale Slope Model

**Figure 3:**
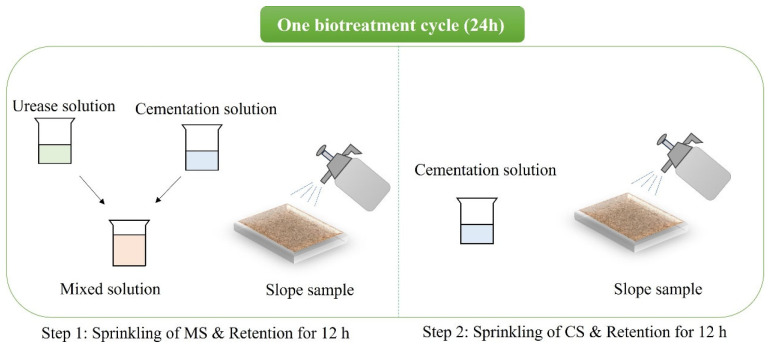
Schematic diagram of one biotreatment cycle for slope model.

The small-scale slope models were prepared in cuboid-shaped molds (16 cm × 10 cm × 3 cm) to achieve an initial dry density of 1.4 g/cm^3^. The biotreatment procedure used in this study was modified from the multiple-phase method proposed by Meng et al. [52]. The PEICP biotreatment for the slope models involved two steps, as shown in Figure 3. Step 1: Equal volumes of soybean crude urease solution (SCUS) and cementation solution (CS) were mixed and applied to the surface of the slope model. The model was then left undisturbed for 12 h to allow the treatment to take effect. Step 2: Only CS was applied to the surface of the slope model to further induce the precipitation of calcium carbonate. The model was again allowed to retain the treatment for 12 h to ensure full biotreatment. For each biotreatment step, the sprinkling solution (either the mixed solution or CS) was applied at a rate of 2 L/m^2^. Once both steps were completed, one biotreatment cycle was considered finished. After the predetermined number of biotreatment cycles, the slope models were dried at room temperature for 6 days. To investigate how the rainfall erosion resistance evolves with bio-cementation level and rainfall intensity, the slope models were prepared for simulated rainfall tests and a series of property tests.

### 2.4. Simulated Rainfall Erosion Test

**Figure 4:**
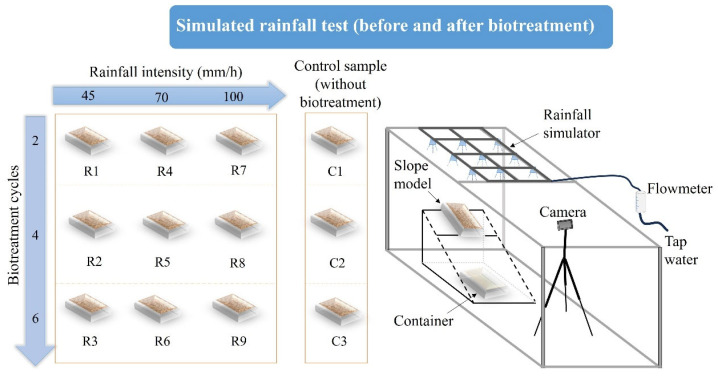
Schematic setup of the simulated rainfall test.

The simulated rainfall tests were conducted in a soil box, as illustrated in Figure 4. Referring to a previous study [66], the slope models were fixed on an inclining metal frame with a tilt angle of 45°. To quantitatively evaluate the soil erosion condition during the slope model being subjected to simulated rainfall, a container was placed below the slope model to collect the wash-out soil. For the biotreated slope models, the wash-out soil was collected and measured every 5 min as the index of soil loss weight. The duration of simulated rainfall for biotreated slope models is 30 min. With regard to control models without biotreatment, the most surface erosion happened within the initial 10 min. Therefore, for control slope models, the collected soil was dried and weighed at 1, 3, 5, 7, 10, 15 min after starting the simulated rainfall test. To provide a certain rainfall intensity, a flowmeter was connected to the rainfall simulator. The rainfall intensities *R*_i_ applied in current study were 45, 70 and 100 mm/h. In front of the slope model, a camera was placed to record the surface erosion pattern.

### 2.5. Properties Tests and Evaluation Method

**Figure 5:**
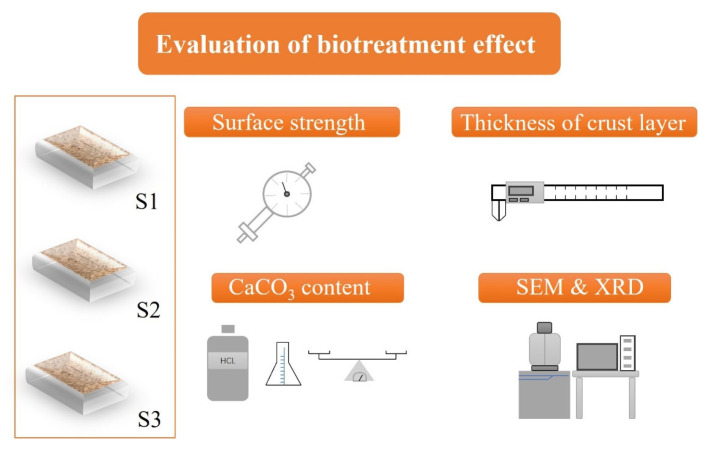
Diagrammatic drawing of property tests.

In order to reveal the fundamental mechanism of surface erosion resistance with a biotreatment approach, a series of property tests were conducted on slope models (S1, S2 and S3), as illustrated in Figure 5. Due to the generation and distribution of calcium carbonate precipitation, bio-cementation effect was introduced into the soil matrix, resulting in the formation of a hard crust layer on the surface of a slope model after biotreatment. The crust layer plays a critical role in promoting the surface rainfall erosion resistance of slope models. Hence, it is vital to evaluate the biotreatment effect by measuring the hardness and thickness of crust layer [41]. Surface strength, as the indicator of hardness, was measured via penetrometer. In current research, five measuring points were chosen to obtain the surface strength of bio-treated slope models. Near the measuring point for surface strength, fragments were collected to measure CaCO_3_ content (*CCC*) by using the acid washing method [21]. In addition, the thickness of crust layer was measured via caliper. To further inspect the inherent mechanism of enhancement in rainfall erosion resistance from the micro perspective, scanning electron microscope (SEM) and X-ray diffraction (XRD) analysis were carried out to identify the microstructure feature and crystal type [67]. The SEM analysis was performed using a ZEISS Sigma 300 (Jena, Germany) scanning electron microscope. Sample preparation: The samples were dried at room temperature for 24 h before gold coating and SEM imaging. The SEM was operated at 200× and 500× magnification to observe the morphology of the calcium carbonate crystals. The XRD analysis was carried out using a SmartLab SE, Rigaku (Tokyo, Japan) diffractometer. The powder diffraction method was used, and samples were ground into a fine powder before being loaded into the XRD machine. The diffraction patterns were recorded in the 2θ range of 4° to 90°. The peaks were identified using HighScore Plus, Version 3.0 and compared with reference patterns from the International Centre for Diffraction Data (ICDD) database. The specific peaks corresponding to calcite were identified and matched with known patterns.

## 3. Results and Discussion

### 3.1. Surface Strength, Calcium Carbonate Content CCC, Thickness of Crust Layer

**Figure 6:**
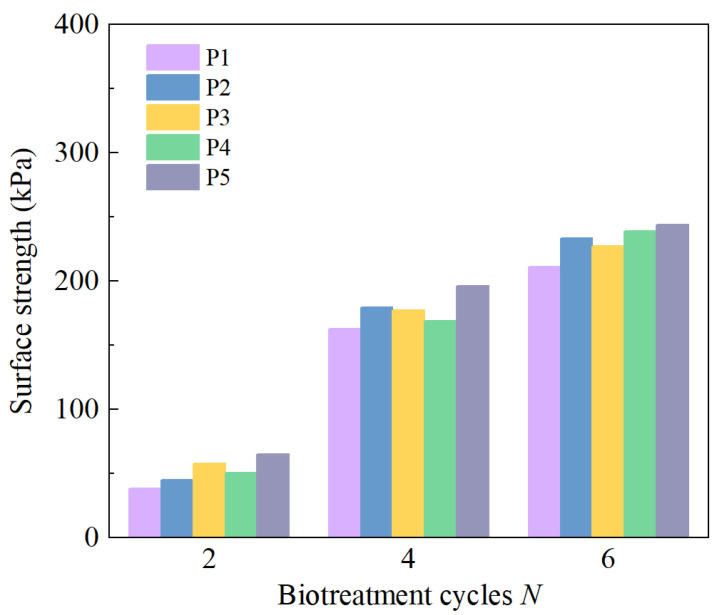
Variation in surface strength with biotreatment cycles.

Generally, surface strength is adopted as the index of assessing the biotreatment effect, especially for the assessment of surface erosion resistance to various environmental factors, such as rainfall, sea wave and wind, etc. [55,68]. Figure 6 illustrates the variation in surface strength in five different measuring points with biotreatment cycles. In answer to increasing biotreatment cycles from two to six, the surface strength of measuring points varies from 38.3 to 244.5 kPa. This is understandable because it has been proved that biotreatment cycle significantly influences the bio-cementation level, which is also reflected in the enhancement of peak stress in triaxial compressive test and unconfined compressive strength (UCS) test in previously reported studies [52,64]. The promotion of mechanical properties mainly comes from the cementation effect introduced by the precipitated calcium carbonate [21]. In the process of soil biotreatment, calcium carbonate precipitation acts as the medium of forming interparticle bonding, resulting in the improvement of surface strength. Figure 6 shows a slight variation in surface strength at different measurement points, suggesting that the biotreatment effect is relatively uniform, achieved through the sprinkling method.

**Figure 7:**
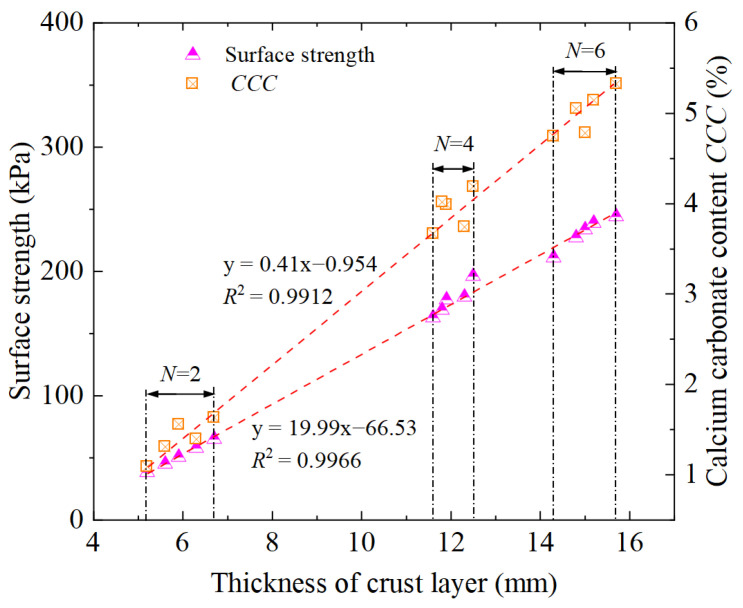
Empirical correlations of surface strength and *CCC* versus thickness of crust layer.

The biotreatment-induced cemented sand layer (crust layer) significantly contributes to enhancing surface erosion resistance to rainfall [4]. In the meantime, bio-cementation level may lead to the distinction in the thickness of crust layer, thereby producing different resistance to surface erosion [14,41,45,46,55]. The influence of biotreatment cycles *N* on the thickness of formed crust layer can be clearly seen in Figure 7, suggesting that increasing the biotreatment cycles *N* greatly facilitate the increase in cemented layer thickness. Figure 7 further revealed the empirical correlations of surface strength and calcium carbonate content *CCC* versus thickness of crust layer. More specifically, the surface strength increased significantly from 38.3 kPa at a crust layer thickness of 5.2 mm (with *N* = 2 cycles) to 244.3 kPa at a crust layer thickness of 15.7 mm (with *N* = 6 cycles). Similarly, the calcium carbonate content (*CCC*) exhibited a notable increase from 1.09048% at a crust layer thickness of 5.2 mm (*N* = 2 cycles) to 5.32381% at a crust layer thickness of 15.7 mm (*N* = 6 cycles). These findings establish a strong positive correlation between crust layer thickness, surface strength, and *CCC*, underscoring the effectiveness of the PEICP method in enhancing erosion resistance.

As shown in Figure 7, it can be observed that there is a positive linear relationship between the surface strength and the thickness of crust layer, similar to observation reported earlier by Wang et al. [69]. Moreover, the positive linear relationship is also capable of depicting the variation of calcium carbonate content *CCC* with the thickness of crust layer. As another important characterization index of biotreatment effect, calcium carbonate content *CCC* was frequently considered in relevant studies involved in the MICP, EICP and PEICP method [38,48,64]. *CCC* reflects the enrichment degree of precipitated calcium carbonate in the biotreated soil matrix from physicochemical properties. It has been reported in previous studies [38,48,54] that increasing the biotreatment cycles can significantly enhance the effect of biotreatment, which also contributes to better performance in bio-cementation depth in current study. Hence, an essential conclusion can be drawn from Figure 7, that is, the increase in biotreatment cycles simultaneously contributes to the increase in surface strength, *CCC* and crust layer thickness.

**Figure 8:**
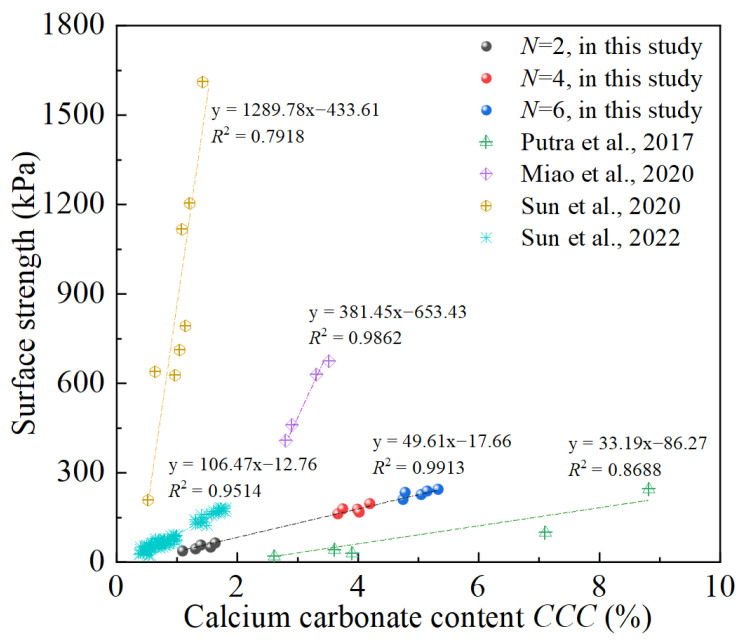
Comparison between current study and prior studies: surface strength versus calcium carbonate content (*CCC*) [41,55,68,70].

As illustrated in Figure 8, comparison between current study and previous studies [41,55,68,70] was carried out in terms of the corresponding relationship between surface strength and calcium carbonate content (*CCC*). A common law can be observed from Figure 8: surface strength increases as *CCC* increases, suggesting that surface strength is positively associated with *CCC*. This is because the cementation effect introduced by biotreatment provides interparticle bonding force that can increase the penetration resistance. High *CCC* level corresponds to preferable biotreatment effect that can restructure the soil matrix and hinder the slight sliding of soil particles along the grain boundaries, thus improving the surface strength [21]. Additionally, linear fitting was performed to characterize the corresponding relationship between surface strength and *CCC*, as shown in Figure 8, where a good match can be observed. By comparison, a relatively large difference in surface strength results can be found in the current study and previous studies conducted by Putra et al. [70], Miao et al. [68] and Sun et al. [41,55]. As described by Meng et al. [52], this can be explained by the influence of many factors on the biotreatment effect, such as soil properties (relative density *D*_r_, particle shape and size), the biological agent properties (source and activity of biocatalyst, composition and concentration of cementation solution) and environmental factors (pH value and temperature), etc.

### 3.2. Visual Observation of Erosion Condition

**Figure 9:**
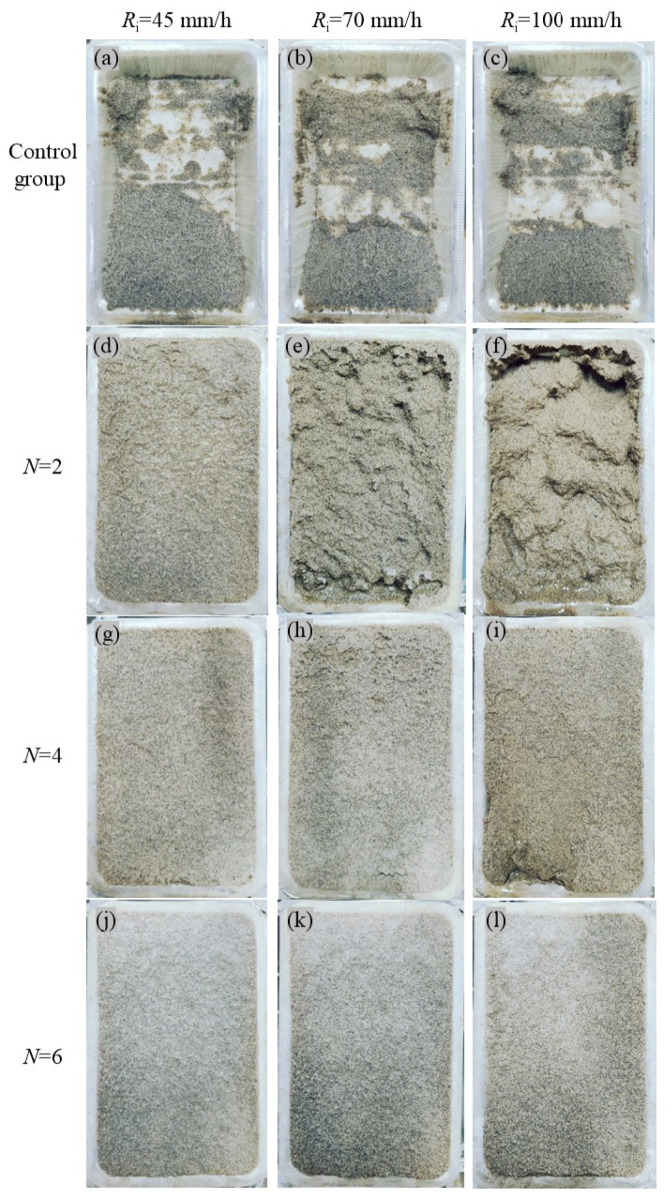
Final surface erosion patterns after 30 min’s simulated rainfall tests: (**a**) C1 under *R*_i_ = 45 mm/h; (**b**) C2 under *R*_i_ = 70 mm/h; (**c**) C3 under *R*_i_ = 100 mm/h; (**d**) R1 under *R*_i_ = 45 mm/h; (**e**) R2 under *R*_i_ = 70 mm/h; (**f**) R3 under *R*_i_ = 100 mm/h; (**g**) R4 under *R*_i_ = 45 mm/h; (**h**) R5 under *R*_i_ = 70 mm/h; (**i**) R6 under *R*_i_ = 100 mm/h; (**j**) R7 under *R*_i_ = 45 mm/h; (**k**) R8 under *R*_i_ = 70 mm/h; (**l**) R9 under *R*_i_ = 100 mm/h.

After the simulated rainfall test, the final surface erosion patterns of slope models showed remarkable diversity, as illustrated in Figure 9. As a whole, the degree of erosion caused by rainfall is positively connected with the rainfall intensity *R*_i_, while the rainfall erosion resistance of slope models enhanced with the improvement of bio-cementation level. Figure 9a–c shows the final surface erosion details of untreated slope models (control group) after simulated rainfall tests of 15 min. It can be seen that at the end of the rainfall simulation experiment, only a small amount of sand was still retained in the mold. Figure 9d–l shows the final morphology of the biotreated slope models after simulated rainfall tests with different rainfall intensities (45, 70 and 100 mm/h) and a duration of 30 min. It can be found that slope models with weak cementation level (*N* = 2) have insufficient resistance to rain erosion, which is more obviously reflected in the surface damage and soil particle erosion condition in simulated rainfall tests with *R*_i_ of 70 and 100 mm/h, shown in Figure 9e–f. Even with regard to *R*_i_ of 45 mm/h, a slight loss of surface soil particles and denudation condition can be observed, as shown in Figure 9d. However, for the slope models with medium bio-cementation level (*N* = 4), the rainfall erosion resistance had been enhanced to a certain extent, reflecting in phenomenon that the slope remained a relatively complete surface after the simulated rainfall test with *R*_i_ of 45 mm/h, as shown in Figure 9g. When the *R*_i_ was increased to 70 mm/h, as illustrated in Figure 9h, slight surface damage occurred and crack appeared in the lower part of the slope model surface after simulated rainfall tests with duration of 30 min, which is consistent with the findings by Jiang et al. [4] and Sun et al. [14]. In Figure 9i, when the *R*_i_ reached 100 mm/h, the slope models with medium bio-cementation level showed obvious erosion damage in the lower part, that is, more soil particles were carried away by the water flow. Raindrops accumulated on the surface of the slope model and formed surface runoff, causing erosion effects on the surface and the washing-off of soil particles [9]. On the other hand, the infiltrated raindrop formed seepage force inside the slope model, giving rise to the dislodging and loss of cemented aggregates. Hence, the erosion damage at the slope toe and the loss of internal soil particles took place under the dual action of the surface runoff erosion force and the internal seepage force. As for the slope models subjected to biotreatment cycles of 6, excellent erosion resistance was displayed during simulated rainfall tests, regardless of rainfall intensity, which can be attributed to the formation and accumulation of calcium carbonate precipitation to restructure the soil matrix, resulting in a bridge effect between adjacent soil particles [21,29,63,65].

**Figure 10:**
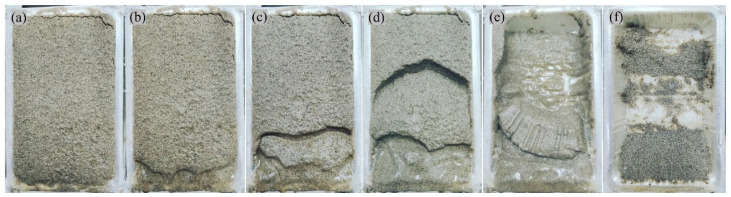
Visual observation of surface erosion process of untreated slope (*R*_i_ = 100 mm/h): (**a**) t = 1 min; (**b**) t = 3 min; (**c**) t = 5 min; (**d**) t = 7 min; (**e**) t = 10 min; (**f**) t = 15 min.

Figure 10 shows the typical erosion process of the untreated slope at 1, 3, 5, 7, 10 and 15 min under *R*_i_ of 100 mm/h. At the beginning of the simulated rainfall test, with the infiltration of rainfall, the moisture content of the slope model gradually increased and the stability of the slope decreased little by little. A tensile crack appeared in the upper part of the slope model, as shown in Figure 10a. As the moisture content of the slope model increases sharply, rainfall infiltration started to slow down, and surface runoff became strong. Subsequently, erosion loss of soil particles began to occur in the lower part of the slope model, where the runoff is most torrential, as shown in Figure 10b. A serious loss of soil particles in the lower part further destabilized the slope, bringing to the surface soil sliding and a step-like collapse in stages, as shown in Figure 10c–e. Finally, it can be seen in Figure 10f that large amounts of soil particles were flushed away by rainfall. Figure 10 mainly demonstrates the erosion process of the untreated slope model under the simulated rainfall test, beginning with surface infiltration and continuing through substantial soil loss and slope destabilization. Similar findings were also reported by Jiang et al. [4], noting the similar erosion processes in sandy-slope surfaces, where initial rainwater infiltration led to surface instability and the loss of soil particles. By providing a more detailed temporal progression of the erosion, this study presented the gradual destabilization of the untreated slope over time.

**Figure 11:**
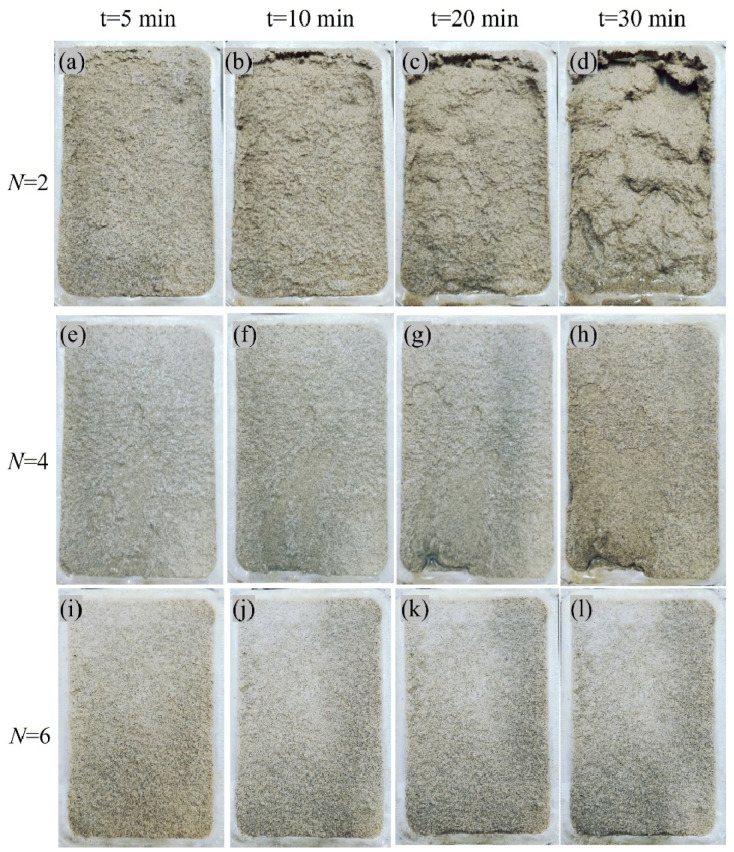
Visual observation of surface erosion at different instants of time (*R*_i_ = 100 mm/h): (**a**) R3, t = 5 min; (**b**) R3, t = 10 min; (**c**) R3, t = 20 min; (**d**) R3, t = 30 min; (**e**) R6, t = 5 min; (**f**) R6, t = 10 min; (**g**) R6, t = 20 min; (**h**) R6, t = 30 min; (**i**) R9, t = 5 min; (**j**) R9, t = 10 min; (**k**) R9, t = 20 min; (**l**) R9, t = 30 min.

Figure 11 shows the erosion details of slopes with different bio-cementation levels during simulated rainfall test (*R*_i_ = 100 mm/h). As can be seen from Figure 11a–d, the erosion process of the weakly bio-cemented slope model (*N* = 2) caused by rainfall progressed from shallow to deep. As the rainfall continues, erosion damage first occurred in the surface layer, and then the internal soil particles were washed away. For the slope model subjected to four biotreatment cycles, it can be seen that, under the dual action of surface runoff erosion and internal seepage force, relatively obvious local erosion damage mainly took place in the lower part of the slope model, where the surface runoff is more likely to accumulate, as shown in Figure 11e–h. Compared with the slope models with biotreatment cycles 2 and 4, the slope models with high bio-cementation level (*N* = 6) did not show obvious erosion damage appearance during the whole simulated rainfall test, indicating that the rainfall erosion resistance was greatly improved by increasing the biotreatment cycle, as evident in Figure 11i–l. Figure 11 mainly presents the effects of varying bio-cementation levels on slope erosion. As rainfall persists, erosion moves from shallow to deeper layers in weakly bio-cemented models (*N* = 2), which is consistent with research by Sun et al. [45], where enzymatic calcification reduced surface erosion and increased resilience to rainfall. In line with Sun et al. [45], who noted a similar erosion pattern under enhanced bio-cementation, this investigation demonstrated that localized erosion damage mostly occurred at the bottom portion of the slope model, where surface runoff collects, when biotreatment cycles were increased to 4. Most remarkably, during the simulated rainfall test, the high bio-cementation slope model (*N* = 6) in this study did not exhibit any discernible erosion damage, highlighting the increased resistance to erosion when the biotreatment cycle is increased. This observation confirms the results of Jiang et al. [4], who found that the microbial biomineralization approach greatly decreased soil erosion by strengthening soil particles, suggesting that bio-cementation is an efficient way to improve slope stability during rainy conditions.

### 3.3. Accumulative Soil Loss Weight and Erosion Rate

**Figure 12:**
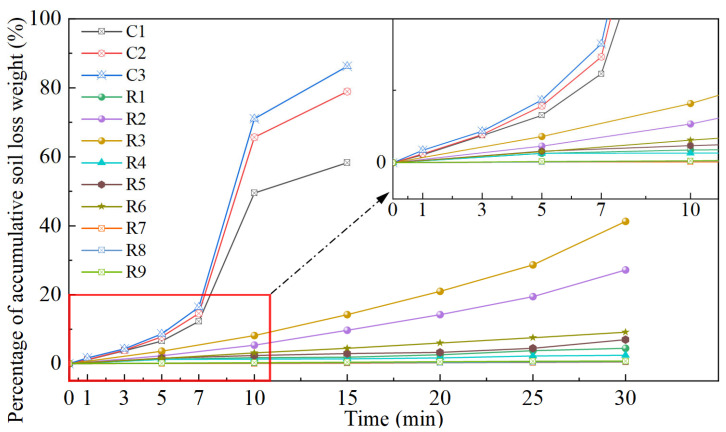
Percentage of accumulative soil loss weight.

The percentage of accumulative soil loss weight could be regarded as the quantification of slope surface erosion. The evolution law of percentage of accumulative soil loss weight with time was characterized in Figure 12. Overall, the appearance of rainfall surface erosion resistance for slopes with different bio-cementation levels embodied clear distinction as for as percentage of accumulative soil loss weight. For the control group (C1, C2 and C3), due to lacking biotreatment, a large amount of soil was washed out when the slopes were exposed to rainfall. By the end of the simulated rainfall tests, the percentage of accumulative soil loss weight for the control group ranged from 58% to 86%. Likewise, for weak bio-cementation level slope models (R2 and R3), the percentage of accumulative soil loss weight progressively increased, which suggested that the integrity of the slope continued to deteriorate during the rainfall with *R*_i_ of 70 and 100 mm/h. For the sample subjected to two biotreatment cycles, the erosion resistance remained inadequate to withstand simulated rainfall intensities of 75 mm/h and 100 mm/h, resulting in cumulative soil particle losses of 27.23% and 41.32%, respectively. However, increasing the number of biotreatment cycles to four effectively controlled the cumulative soil particle loss to within 10%. A rather severe soil loss condition appeared in the slope model R6 under *R*_i_ of 100 mm/h. Furthermore, the erosion condition of slope models (R1, R4 and R5) is mild, which can be attribute to relatively low *R*_i_ of simulated rainfall test or comparatively high bio-cementation level of slope model. Further enhancing the biotreatment to 6 cycles resulted in the bio-treated slope achieving optimal resistance to rainfall-induced erosion, maintaining its structural integrity throughout the tests. Slope models (R7, R8 and R9) maintain a preferable integrity until the termination of simulated rainfall test, which was reified as relatively flat curves in Figure 12. In summary, the law reflected in the percentage of accumulative soil loss weight with time is in line with the phenomenon observed in Figure 9, Figure 10 and Figure 11.

**Figure 13:**
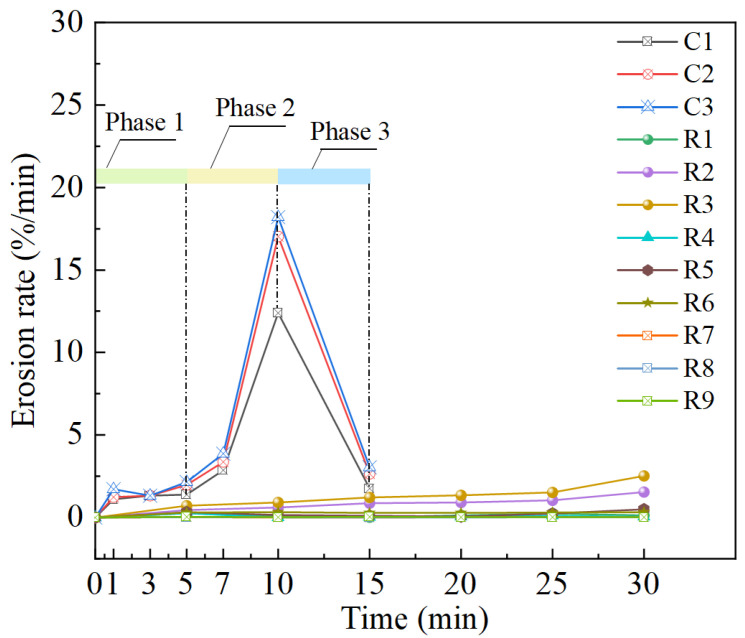
Erosion rate of biotreated and untreated slope.

Figure 13 further explored the erosion rate under different test conditions. Broadly speaking, there are mainly three laws in terms of the erosion rate: (1) fluctuate pattern (control group C1, C2 and C3); (2) growth pattern (R2 and R3); and (3) stable pattern (other slope models). The fluctuate feature can be clearly seen in Figure 13, which also embodies the three phases existing in the erosion rate curves of the control group. In the initial stage of simulated rainfall test (phase I), the infiltration of rainfall led to an increase in moisture and a decrease in slope stability. At this phase, the erosion is mainly caused by the loss of surface soil particles, and the erosion rate increased slowly. The erosion rate developed very quickly from 5 min and reached a peak at 10 min, indicating that the slope stability deteriorated sharply and soil particles were rapidly flushed away. This phenomenon in phase II can be attributed to the coupled influence of surface runoff and internal seepage force, which induces the quick increase in erosion rate. The erosion rate dramatically declined in phase III, suggesting that the soil loss principally accomplished in phase I and II. For slope models (R2 and R3), the erosion rate curves showed the characteristic of growth pattern, indicating that the erosion condition of the slope became progressively worse with the simulated rainfall test being conducted. After biotreatment, the crust layer primarily takes the function of resisting surface erosion [4,14,41,45,46,55]. This means that, when breakage appears on the crust layer because of rain-wash, the erosion resistance of the biotreated slope will gradually degrade. Meanwhile, the erosion rate curves of the slope models (R1, R4–9) remained relatively stable during the simulated rainfall test on account of low *R*_i_ or high bio-cementation level.

### 3.4. Analysis of Microscopic Mechanism

**Figure 14:**
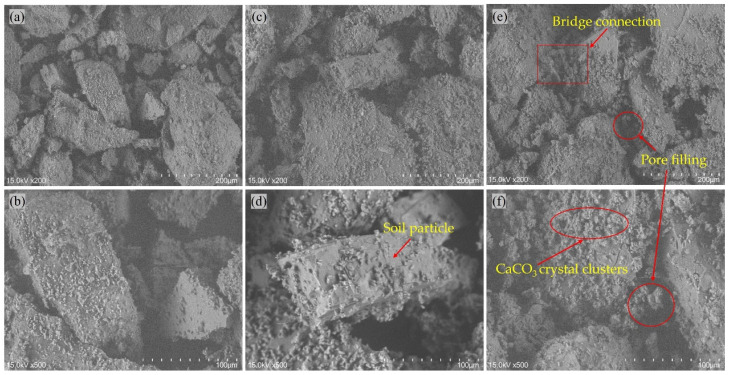
SEM images of biotreated slope models with different biotreatment cycles: (**a**) *N* = 2, magnification = 200; (**b**) *N* = 2, magnification = 500; (**c**) *N* = 4, magnification = 200; (**d**) *N* = 4, magnification = 500; (**e**) *N* = 6, magnification = 200; (**f**) *N* = 6, magnification = 500.

**Figure 15:**
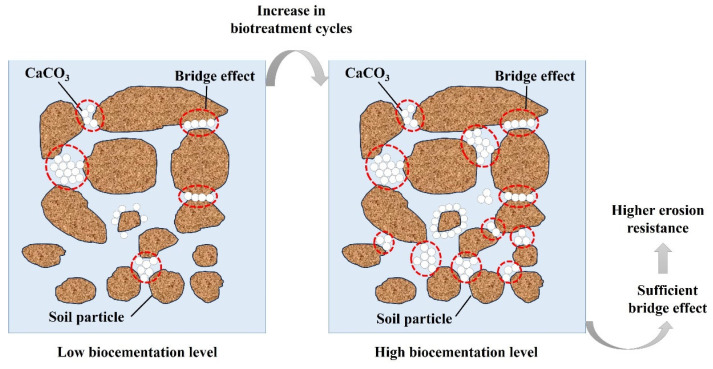
The microcosmic mechanism of the influence of bio-cementation level on rainfall erosion resistance.

**Figure 16:**
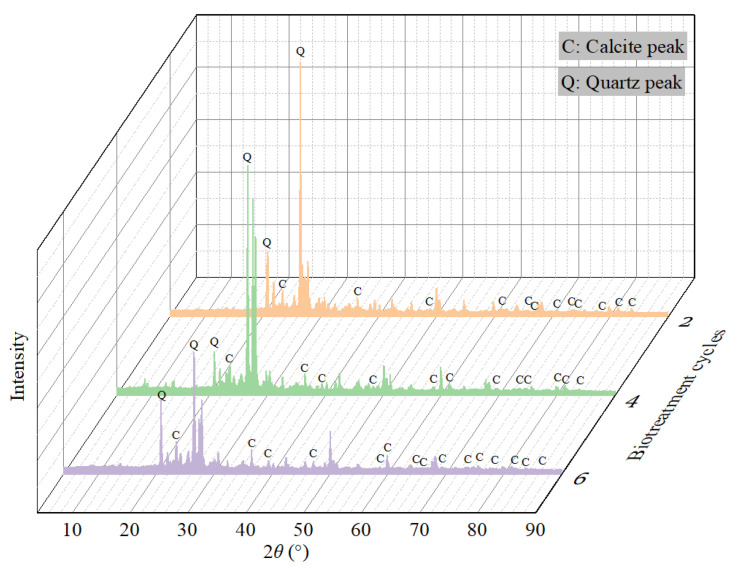
XRD analysis of biotreated models with different biotreatment cycles.

To reveal the mechanism for rainfall erosion resistance of slope model after biotreatment, scanning electron microscope (SEM) tests and X-ray diffraction (XRD) analysis were performed on the slope models with different biotreatment cycles. The microstructure of surface specimens with magnification of 200 and 500 was illustrated in Figure 14, which clearly reflects the bio-cementation level and crystal morphology. Visible differences were observed among three different bio-cementation level specimens. For biotreated slope model with biotreatment cycle 2, only tiny CaCO_3_ crystals deposited on the surface of sand particles, as shown in Figure 14a,b. When the biotreatment cycle was increased to four cycles, bigger size and moderate amount of CaCO_3_ crystals was observed in Figure 14c,d Nonetheless, it should be noted that the bridge effect between neighboring soil particles is not sufficient. By comparison, when further increasing biotreatment cycles to six, CaCO_3_ precipitation in form of crystal clusters distributed on the surface of soil particles and filled the void space, as shown in Figure 14e,f. More importantly, the agglomeration and deposition of crystal clusters at the connecting point of soil particles formed an effective bridge-connection between adjacent particles, thereby generating strong bonding force. As indicated by Cheng et al. [71] and Jiang et al. [4], there is a competitive relationship between nucleation and crystal growth during CaCO_3_ precipitation. By increasing the biotreatment cycles, longer growth periods and more sufficient chemical supplements were provided to facilitate the crystal growth during the nucleation process. Furthermore, greater size and more amounts of CaCO_3_ crystal clusters are more liable to constitute an effective bridge-connection [52], as shown in Figure 15. Thus, the slope models with highest bio-cementation level (*N* = 6) showed optimum erosion resistance to rainfall due to the strong bonding force existing between the soil particles. In Figure 16, via XRD analysis, the mineralogical composition of generated CaCO_3_ crystals was affirmed as calcite, which has better thermodynamical stability than aragonite and vaterite. In addition, it is crucial that CaCO_3_ crystals in the form of calcite possess superb mechanical property and can solidify the skeleton of soil particles [72]. In addition, it is worth noting that the influence of biotreatment cycles on crystallinity can be clearly seen in Figure 16. More specifically, increasing biotreatment cycles can achieve superior crystallinity, which is comprehensively reflected as the amounts and intensity of diffraction peaks of calcite.

### 3.5. Shortcomings and Application Prospects

Even though biomineralization technology has shown significant advantages in enhancing slope erosion resistance, there are still many key problems to be solved in its large-scale engineering application. First, there is uncertainty about the long-term durability of bio-basing layer in complex natural environments. Under the action of freeze–thaw cycle and alternating dry and wet environments, there might be micro-cracks in the bio-cemented layer, leading to structural weakening and shear strength reduction. At the same time, the inherent microbial activity in the soil may degrade the cementing matrix, and these factors will affect the durability of the treatment effect. When it comes to engineering execution, there are technical obstacles that prevent large-scale slopes from receiving consistent biotreatment. Three limitations are the primary cause of this: First, the distribution of urease in soil is difficult to control accurately, resulting in spatial heterogeneity of cementation reaction; Second, there is a significant difference in the penetration of substrate solution in heterogeneous soil, which affects the reaction uniformity. Thirdly, the differences in mineral composition, pore structure, pH value and other physical and chemical properties of soil in different regions will significantly affect the bio-cementation effect.

In order to promote the practical application of this technology, more attention should be paid to the following aspects: improve the environmental stability of the bio-cemented layer by introducing an organic modifier; optimize the processing parameters to achieve uniform biotreatment effect; carry out long-term field monitoring and establish an environmental impact assessment system. Only by systematically addressing these key issues can biomineralization technology truly become an effective solution for sustainable erosion control.

## 4. Conclusions

In the current study, to investigate the feasibility of enhancing slope surface rainfall erosion resistance via PEICP approach, simulated rainfall erosion tests considering different bio-cementation levels (biotreatment cycles) and rainfall intensity *R*_i_ were performed. The following conclusions can be drawn from this study:

1. By increasing biotreatment cycles, higher bio-cementation level and superior biological treatment effect can be achieved, contributing to the increase in surface strength, *CCC* and crust layer thickness, which is beneficial for rainfall erosion resistance. Specifically, surface strength increased from 38.3 kPa at a crust layer thickness of 5.2 mm (*N* = 2 cycles) to 244.3 kPa at a crust layer thickness of 15.7 mm (*N* = 6 cycles). Calcium carbonate content (*CCC*) increased from 1.09% at a crust layer thickness of 5.2 mm (*N* = 2 cycles) to 5.32% at a crust layer thickness of 15.7 mm (*N* = 6 cycles).
2. Coupled effects of surface runoff erosion force and the internal seepage force triggered the surface erosion and internal soil particle loss of slope. For the untreated group and weakly bio-cemented slope (*N* = 2), the percentage of accumulative soil loss weight ranged from 58% to 86% by the end of the simulated rainfall tests. For the sample biotreated two times, its erosion resistance was still insufficient to cope with the simulated rainfall intensities of 75 mm/h and 100 mm/h, with cumulative soil particle losses reaching 27.23% and 41.32%, respectively. When crustal erosion occurs, the erosion rate accelerates. When the number of treatments was increased to four, the cumulative loss of soil particles was controlled within 10%. Finally, when the biotreatment cycles reached six cycles, the biotreated slope demonstrated optimal resistance to rainfall-induced erosion and remained intact.
3. SEM images indicated that increasing the biotreatment cycle facilitated the crystal growth and formed larger size crystal clusters, which are more likely to bridge-connect adjacent soil particles. The bridge-connection effect induced by CaCO_3_ precipitation provided a strong bonding force that played a vital role in enhancing the erosion resistance to rainfall. Furthermore, the mineralogical composition of generated CaCO_3_ crystals was affirmed as calcite, which has better mechanical properties.

## Figures and Tables

**Table 1:** Basic properties of *K-7* sand.

Index	*K-7* Sand
Specific Gravity of Soil Particles, Gs	2.67
Unit weight, γt (kN/m^3^)	14.8
Coefficient of uniformity, Cu	4
Coefficient of curvature, Cc	1.21
Maximum density, ρmax (g/cm^3^)	1.63
Minimum density, ρmin (g/cm^3^)	1.27
Maximum void ratio, emax	1.202
Minimum void ratio, emin	0.641

**Table 2:** Slope model arrangement for simulated rainfall tests and property tests.

Model	Test Conditions		Test Category
	Biotreatment Cycles *N*	Rainfall Intensity *R*_i_	
S1	2	——	Property tests
S2	4	——	
S3	6	——	
C1	0	45	Control group (without biotreatment)
C2	0	70	
C3	0	100	
R1	2	45	Rainfall erosion analysis (after biotreatment)
R2	2	70	
R3	2	100	
R4	4	45	
R5	4	70	
R6	4	100	
R7	6	45	
R8	6	70	
R9	6	100	

## Data Availability

The original contributions presented in this study are included in the article.
